# Microbial electrochemical enhanced composting of sludge and kitchen waste: Electricity generation, composting efficiency and health risk assessment for land use

**DOI:** 10.1016/j.heliyon.2024.e35678

**Published:** 2024-08-05

**Authors:** Tengteng Hu, Yunhan Lin, Yingyu Liu, Qingliang Zhao, Hang Yu, Zhugen Yang, Fanyu Meng

**Affiliations:** a Department of Environmental Hygiene, School of Public Health, Harbin Medical University, Harbin, 150081, China; b State Key Laboratory of Urban Water Resources and Environments (SKLUWRE), Harbin Institute of Technology, Harbin, 150090, China; c Collaborative Innovation Center for Vessel Pollution Monitoring and Control, Dalian Maritime University, Dalian, 116026, China; d School of Water, Energy and Environment, Cranfield University, Cranfield, MK43 0AL, United Kingdom

**Keywords:** Microbial electrochemical enhanced compost, Dewatered sludge, Kitchen waste, Land use, Health risk assessment

## Abstract

To realize the energy and resource utilization from organic solid waste, a two-phase microbial desalination cell (TPMDC) was constructed using dewatered sludge and kitchen waste as the anode substrate. The performance of electricity generation and composting efficacy was investigated, along with a comprehensive assessment of the potential health risks associated with the land use of the resulting mixed compost products. Experimental outcomes revealed a maximum open-circuit voltage of 0.893 ± 0.005 V and a maximum volumetric power density of 0.797 ± 0.009 W/m³. After 90 days of composting enhanced by microbial electrochemistry, a significant organic matter removal rate of 31.13 ± 0.44 % was obtained, and the anode substrate electric conductivity was reduced by 30.02 ± 0.04 % based on the anode desalination. Simultaneously, there was an increase in the content of available nitrogen, phosphorus, and potassium, as well as an improvement in the seed germination index. The forms of heavy metals shifted from bioavailable to stable residual states. The non-carcinogenic hazard index (*HI*) values for heavy metals and polycyclic aromatic hydrocarbons (PAHs) during the land use of compost products were less than 1, and the total carcinogenic risk (*TCR*) values for heavy metals and PAHs were below the acceptable threshold of 10^−4^. The occupational population risk of infection from five pathogens was higher than that of the general public, with all risk values ranging from 8.67 × 10^−8^ to 1, where the highest risk was attributed to occupational exposure to *Legionella*. These outcomes demonstrated that the mixture of dewatered sludge and kitchen waste was an appropriate anode substrate to enhance TPMDC stability for electricity generation, and its compost products have promising land use suitability and acceptable land use risk, which will provide important guidance for the safe treatment and disposal of organic solid waste.

## Highlights

• Kitchen waste improved the microbial electrochemical compost performance of sludge.
• Land use feasibility of mixed organic solid waste compost was improved.
• The health risk values of compost product land use are within acceptable ranges.

## 1 Introduction

The globe currently confronts the dual challenges of municipal organic solid waste management and soil degradation. Dewatered sludge, a primary component of municipal organic solid waste, has seen its production escalate with the rapid pace of urbanization. In China, the output of dewatered sludge surpassed 65 million tons in 2020 and is projected to exceed 90 million tons by 2025 [1]. Kitchen waste, another abundant type of organic solid waste, was produced over 120 million tons in China in 2021 [2]. Both dewatered sludge and kitchen waste are rich in organic matter and nutrients, which positions them as viable composting materials to rehabilitate degraded soil and foster crop growth [3,4]. Nevertheless, applying untreated organic waste directly to soil can hinder plant growth, as the rapid increase of small organic molecules may compete with plant roots for oxygen during the microbial decomposition process Researches by Oleszczuk et al. [5] and Ramírez et al. [6] have indicated that sludge could dramatically impair the growth and development of plants and could even inhibit soil invertebrate development [[7], [8], [9]]. Moreover, dewatered sludge contains pathogenic organisms and potentially toxic components [10,11], which pose a significant risk to soil ecosystem health. Heavy metals, including Cr, Ni, Cd, Pb, which are classified as toxic, are common contaminants in dewatered sludge [[12], [13], [14]]. Polycyclic aromatic hydrocarbons (PAHs), a group of highly carcinogenic, mutagenic, and toxic organic pollutants [15], are also prevalent in dewatered sludge [16]. Prolonged exposure to PAHs has been linked to an increased risk of diseases such as lung, bladder, and skin cancer [[17], [18], [19]]. The introduction of raw dewatered sludge to soil can introduce heavy metals and PAHs, posing a threat to human health through various exposure routes, including inhalation, ingestion, and dermal contact during land use [20]. Furthermore, pathogenic microorganisms that concentrate in sludge during the wastewater treatment can evade disinfection processe, presenting occupational and public health risks due to their potential to disperse through aerosols [21,22]. Kitchen waste, notable for its perishable nature, strong odors, and tendency to harbor harmful parasites, pathogens, and mycotoxins [23], is traditionally disposed of in landfills, incinerated, or used as feedstock. These practices contribute to increased resource consumption, environmental pollution, and the spread of human diseases if not properly treated [24,25]. Overall, nutrient imbalance, pathogenic organisms, toxic substances, foul odors, and high soluble salt levels can negatively impact soil properties, thereby impeding the beneficial utilization of organic waste [[26], [27], [28], [29]]. Therefore, the necessity for further treatment of organic solid waste before land use is imperative.

Composting serves as a prevalent method for treating organic solid waste, achieving a degree of reduction, stabilization, and harmlessness. Aerobic composting of organic solid waste offers higher stability but at a higher energy cost, while anaerobic composting is energy-efficient but results in less stable products [30]. Consequently, there is an urgent need for meticulous research to develop multifunctional and highly efficient composting technologies to meet the growing demand for organic solid waste treatment.

In recent years, microbial electrochemical systems (MES) have been explored to enhance the degradation efficiency of organic solid waste and enable simultaneous energy recovery [31]. Notably, the invention of solid phase microbial fuel cell (SPMFC) and compost microbial fuel cell (CMFC) achieved more efficient composting. Yu et al. [32] confirmed that the degradation of sludge was speeded up by the bioelectricity generation when the anode chamber of microbial fuel cell (MFC) was utilized for treating sewage and dewatered sludge. Wang et al. [33] employed SPMFC to shorten the composting process of agricultural solid wastes. Khudzari et al. [34] used a mixture of fruits, vegetables and soil as CMFC substrate, enhancing the degradation efficiency of organic matter by 6 %–8 %. However, there are still some challenges in MFC enhanced organic solid waste composting, including lower electricity production efficiency, questionable stability of end product, and anode substrate salinization.

Two-phase microbial desalination cell (TPMDC), which is derived from the MFC by inserting a desalination chamber and concentration chamber between the cathode and anode, was developed to address the problem of anode substrate salinization, serving as a sustainable approach for desalination and concurrent organic solid waste treatment. The ion migration in the desalination system is driven by the bioelectric potential difference. Cations in the anode chamber are transferred to the concentration chambers through cation exchange membranes, which achieves the desalination of anode substrates. Salinity in the desalination chamber are removed through the migration of anions and cations to the concentration chambers and cathode chambers, respectively [35]. This two-phase desalination is not only benefited land use of anode sludge, but also can be used as a pretreatment for subsequent reverse osmosis (RO) processes to reduced desalination costs [[36], [37], [38]]. Additionally, integrating the desalination function into the MFC significantly improved the energy production performance [39]. Meng et al. [40] used dewatered sludge as the TPMDC anode substrate, achieving an anode sludge desalination rate of 31.63 ± 1.26 %, a maximum output power of 3.178 W/m^3^, and an organic matter removal rate of 25.71 ± 0.15 % over 200 days of operation. The available nutrient contents in the anode sludge increased, and heavy metals were transformed into stable states.

In summary, although microbial electrochemical systems enhance sludge maturity, making it suitable for subsequent land use, issues persist, including reduced microbial activity and slower organic matter degradation due to low C/N ratio of dewatered sludge during the composting process, as well as an insufficient risk assessment of its land use. Therefore, it is of great significance to select appropriate co-composting substrate to improve the performance of electricity production, such as kitchen waste, which can provide abundant carbon source for microbial activities [41,42]. Furthermore, conducting a comprehensive health risk assessment of compost products for land use is of paramount importance.

The primary objectives of this study are to (1) construct a mixed composting system of dewatered sludge and kitchen waste enhanced by microbial electrochemical system; (2) analyze the power production performance and composting efficiency of the system; (3) assess health risk of compost products for land use thoroughly, including heavy metals and PAHs from three exposure scenarios (ingestion, inhalation, and dermal contact), and pathogenic microorganisms from the exposure of occupational and general populations.

## 2 Materials and methods

### 2.1 Experimental reactor and materials

**Fig. 1:**
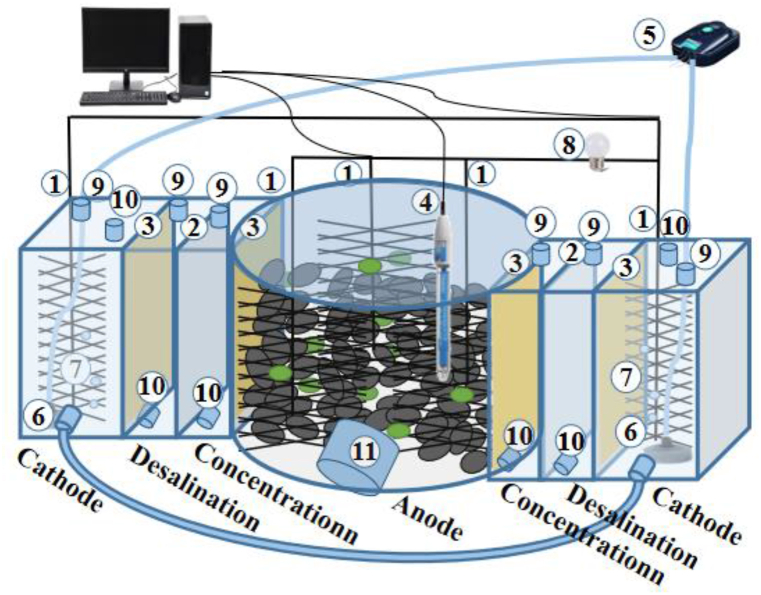
Construction of two-phase microbial desalination cell (1) Carbon fiber brushes; (2) Anion-exchange membranes (AEM); (3) Cation-exchange membranes (CEM); (4) Ag/AgCl reference; (5) Air pump; (6) Aerator; (7) Air bubbles; (8) External resistance; (9) Inlet; (10) Outlet; (11) Sampling point.

The construction of TPMDC adhered to the our previous approach [40], as depicted in Fig. 1. The TPMDC featured a symmetrical design, central to which was a cylindrical anaerobic anode chamber with dimensions of 6 cm in radius and 13 cm in height. Flanking this chamber were orderly arranged cubic structures: concentration chambers (length 9 cm × width 3 cm × height 9 cm), desalination chambers (length 9 cm × width 3 cm × height 9 cm), and cathode chambers (length 9 cm × width 5 cm × height 9 cm). Cation exchange membranes (CEM, Ultrex CMI- 7000, 10 cm × 10 cm) were strategically employed to separate the anode chamber and the concentration chambers, as well as the cathode chambers and the desalination chambers. Separation between the concentration and desalination chambers was achieved using anion exchange membranes (AEM, Ultrex AMI-7001, 10 cm × 10 cm). The anode electrode was a 9 cm diameter carbon fiber brush (STS40 24 K, Toho Tenax), while the cathodic electrode was a composite material consisting of carbon brushes integrated with graphite particles (diameter of 1–5 mm, Jiuxin Carbon Goods Co., Jilin, China).

The dewatered sludge was sourced from a municipal wastewater treatment plant located in Harbin. The kitchen waste, a blend of various food items including rice, dumplings, vegetables, cooked meat, mushrooms, and potatoes, was processed by blending with sterile deionized water in a mixer to serve as the anode substrate conditioner. The experimental soil for land use was from the Songnen plain, characterized by a pH of 9.37 ± 0.21, a soil alkalization degree of 39.38 ± 2.52 %, which refers to the proportion of exchangeable sodium content to the total cation exchange capacity, and an organic matter content of 2.91 ± 0.16 %. The bulk density of the anode compost product and the experimental soil were recorded as 1.124 ± 0.011 g/cm^3^ and 2.565 ± 0.012 g/cm^3^, respectively. A detailed overview of the experimental materials is presented in Table 1.

**Table 1:** The properties of experimental materials.

name	water content (%)	organic matter (%)	C/N
DS	70.46 ± 1.21	48.38 ± 1.25	7.52 ± 0.15
KW	90.42 ± 0.98	81.14 ± 1.09	24.33 ± 0.23
DS + KW	74.08 ± 1.07	54.93 ± 1.06	10.88 ± 0.37
experimental soil	14.01 ± 0.12	2.91 ± 0.16	20.04 ± 0.25

Note: DS: dewatered sludge; KW: kitchen waste.

### 2.2 Experimental methods and sample collection

#### 2.2.1 Experimental methods

The procedures for anode and cathode electrode treatment, catholyte preparation, inoculation, replenishment, and replacement, as well as the preparation and replacement of salt solution in both desalting chambers and concentration chambers, were executed in accordance with the established methods outlined in a previous study [43]. Based on the intention of utilizing sludge as the principal composting material and the results of anaerobic co-digestion of sludge and kitchen [[44], [45], [46]], a mixture of dewatered sludge and kitchen waste with a mass ratio of 4:1 was introduced into the anode chamber of TPMDC. The anode functioned in an anaerobic mode throughout the entire experimental cycle. After 90 days of continuous operation at ambient temperature, the anode compost products were employed to amend the experimental soil, which had been ground and sieved through a 1 mm standard mesh. The soil was amended at a dilution ratio of 1.75 × 10^−3^ (implying that 1 g of anode compost products and soil mixture contains 0.00175 g of dry compost products. This equates to an application rate of 30.75 tons of anode compost products with a water content of 74 % per hectare, applied to a depth of approximately 15 cm). The improved soil was then transferred to pots with dimensions of 15 cm in diameter and 20 cm in height for incubation under controlled conditions: a temperature of 25 ± 2 °C, a relative humidity of 70 ± 2 %, and a photo period of 14 h of light per day.

#### 2.2.2 Sample collection and analysis method

The full voltage, cathode and anode electrode potential against the Ag/AgCl reference electrode (+197 mV vs. standard hydrogen electrode, SHE, model 218, Leici, Shanghai, China) were continuously recorded using a multi-channel voltage collector (PISO-813, ICP DAS, Co., Ltd, Beijing, China), and the averages were calculated per 24 h. The volumetric power density was calculated and normalized based on the fixed external resistance (R = 1000 Ω) and the net anode chamber (NAC) volume. Polarization curves with the estimation of internal resistance (*R*_int_) and the maximum power density (*P*_max_) curves were depicted according to previously described [47].

Anode substrate samples were collected every five days in the following way: anode substrate was taken out from three layers and mixed evenly into one sample for physicochemical properties analysis. The mixed anode compost samples were diluted 10 times with sterile deionized water and centrifuged at 4000 rpm for 20 min, the supernatants were measured for electrical conductivity (EC) using a conductivity meter (model DDSJ-308A, Leici, Shanghai, China). The same anode compost samples were dried to constant weight in a constant temperature drying oven (40 ± 2 °C), ground and sieved (0.150 mm standard screen) for analysis of organic matter, available nitrogen, phosphorus and potassium. The analysis methods of these indicators are referred to the “Analytical methods for soil and agro‐chemistry (3rd edition)” [48], specifically, using potassium dichromate method-external heating method for organic matter measurement, alkaline hydrolysis diffusion method for available nitrogen, Mo–Sb antispetrophotography method after 0.5 mol L^−1^ NaHCO_3_ leaching for available phosphorus, and flame photometry after l.0 mol L^−1^ NH_4_OAC leaching for available potassium. In addition, total carbon and nitrogen, which are used to calculate C/N ratio, was measured by Elemental analyzer (model Vario EL, Elementar, Germany). All the analyses were made at least 3 times and the average values with standard deviations were presented. The germination index (GI) of pakchoi seeds was assessed according to the method previously reported [49].

Heavy metals and metalloids content in anode substrate and improved soil were determined by mixed acid microwave digestion and ICP-AES method [40], including As, B, Cd, Cr, Cu, Hg, Ni, Pb, Zn. The chemical forms of heavy metals were analyzed using sequential extraction procedure proposed by the European Standard, Measurements and Testing (SM&T) program, formerly the Community Bureau of Reference (BCR) [50]. The PAHs were detected using gas chromatography-mass spectrometry of Chinese standards method (HJ 805–2016) [51]. Then the contents of heavy metals and PAHs in the improved soil after incubation of 30 days were used to calculate the health risks of anode compost land use. The anode substrates from different periods were directly sealed in a 10 ml sterile centrifuge tubes, stored on dry ice and used for the subsequent macro-genomic analyses. According to the prediction of gene number, the abundance of pathogenic microorganisms and the amount of anode substrates land use, the concentrations of five pathogenic microorganisms (including *Escherichia_coli*, *Salmonella*, *Vibrio_cholerae*, *Legionella*, and *Shigella*) in the soil and aerosols during the land use were estimated.

### 2.3 Health risk assessment model of heavy metals and PAHs

Risk assessment is a comprehensive, multi-step process that encompasses hazard identification, exposure assessment, dose-response assessment and risk characterization [52]. The objective of human health risk assessment is to quantify the potential health risk and the likelihood of adverse health effects stemming from exposure to chemicals in an environmentally compromised setting. For the present study, the health risk assessment model recommended by the USEPA was employed to estimate both non-carcinogenic and carcinogenic risks for adults and children associated with heavy metals and PHAs present in the soil after amendment. Multiple exposure pathways were considered, including ingestion, dermal contact, and inhalation.

The study calculated the hazard quotient (*HQ*), total non-carcinogenic hazard index (*HI*), carcinogenic risk (*CR*), and total carcinogenic risk (*TCR*) for the heavy metals and PHAs in the improved soil. The selection of variable parameters was informed by the technical guidelines for risk assessment of soil contamination of land for construction in China [53] and the Chinese Population Exposure Parameters Manual [[54], [55], [56]]. The heavy metals under investigation included arsenic (As), cadmium (Cd), chromium (Cr), nickel (Ni), copper (Cu), mercury (Hg), lead (Pb), and zinc (Zn). The PAHs encompassed benzo[a]anthracene (BaA), chrysene (Chr), benzo[k]fluoranthene (BkF), benzo[b]fluoranthene (BbF), indeno[1,2,3-c,d]pyrene (IcdP), dibenzo[a,h]anthracene (DahA), naphthalene (Nap), acenaphthene (Ace), acenaphthylene (Acy), fluorene (Flu), phenanthrene (Phe), anthracene (Ant), fluoranthene (Fla), pyrene (Pyr), and benzo[g,h,i]perylene (BghiP). The concentration of these heavy metals and PAHs in the soil subsequent to the application of anode compost products was shown in Table 2. The pertinent calculation equations were as follows:

**Table 2:** The contents of chemical pollutants in soils.

heavy metals	measured values (mg/kg)	reference values (mg/kg)	PAHs	measured values (mg/kg)	reference values (mg/kg)
As	7.73 ± 1.01	20	NaP	0.74 ± 0.05	25
Cd	0.79 ± 1.34	20	Ace	0.13 ± 0.02	5.0
Cr	66.59 ± 2.51	400	Acy	0.08 ± 0.02	5.0
Cu	66.38 ± 2.67	2000	Flu	0.42 ± 0.07	5.0
Hg	0.62 ± 0.01	8	Phe	1.96 ± 0.23	5.0
Ni	20.766 ± 1.45	150	Ant	1.85 ± 0.17	5.0
Pb	26.53 ± 3.63	400	Fla	1.21 ± 0.09	5.0
Zn	211.41 ± 7.87	500	Pyr	0.88 ± 0.04	5.0
			BaA	0.45 ± 0.05	5.5
			Chr	0.41 ± 0.03	490
			BkF	0.53 ± 0.07	55
			BbF	0.39 ± 0.04	5.5
			IcdP	0.09 ± 0.02	5.5
			DahA	0.04 ± 0.01	0.55
			BghiP	0.26 ± 0.03	5.0

Note: mg/kg was calculated based on dry basis.

The reference value is based on the “Environmental quality standards for soils (Revised)” (GB15618—2008) and the “Soil environmental quality-risk control standard for soil contamination of development land (Implementation)” (GB36600—2018).

The average daily dose (ADD, mg/kg/day) by inhalation *(ADD*_*inh*_), dermal contact (*ADD*_*der*_), and ingestion (*ADD*_*ing*_) were calculated as the following equations (1), (2), (3):

(1) $${ADD}_{inh}=\frac{C\times InhR\times EF\times ED}{PEF\times BW\times AT}$$

(2) $$ADD_{der}=\frac{C\times SA\times AF\times ABS\times EF\times ED\times CF}{BW\times AT}$$

(3) $${ADD}_{ing}=\frac{C\times IngR\times EF\times ED\times \text{CF}}{BW\times AT}$$

where the *C* was the measured concentration of heavy metals and PAHs in the soil (mg/kg), and the remaining parameters and their values were detailed in Table 3.

**Table 3:** The exposure parameters for risk assessment.

name of the parameter	value
exposure duration (*ED*, year)	30 (a)
	6 (c)
exposure frequency (*EF*, d/year)	300
absorption factor (*ABS*)	0.001
body weight (*BW*, kg)	60 (a)
	15 (c)
average time (*AT*, d)	25550
ingestion rate (*IngR*, mg/d)	100 (a)
	200 (c)
inhalation rate (*InhR*, m^3^/d)	20 (a)
	5 (c)
exposed skin area *(SA*, cm^2^)	5700 (a)
	1600 (c)
skin adherence (*SL*, mg/cm^2^·d)	0.07 (a)
	0.2 (c)
particle emission factor (*PEF*, m^3^/kg)	1.36 × 10^9^

Note: a: adults; c: children.

The hazard quotient (*HQ*) by inhalation *(HQ*_*inh*_), dermal contact (*HQ*_*der*_), and ingestion (*HQ*_*ing*_) and total non-carcinogenic hazard index (*HI*) were calculated according to Eqs (4), (5), (6), (7). The carcinogenic risk (*CR*) by inhalation *(CR*_*inh*_), dermal contact (*CR*_*der*_), and ingestion (*CR*_*ing*_), and total carcinogenic risk (*TCR*) were calculated according to Eqs (8), (9), (10), (11).

(4) $${HQ}_{inh}=\frac{{ADD}_{inh}}{{RfD}_{inh}}$$

(5) $${HQ}_{der}=\frac{{ADD}_{der}}{{RfD}_{der}}$$

(6) $${HQ}_{ing}=\frac{ADD_{ing}}{{RfD}_{ing}}$$

(7) $$HI={HQ}_{inh}+{HQ}_{der}+{HQ}_{ing}$$

(8) $${CR}_{inh}={ADD}_{inh}\times {SF}_{inh}$$

(9) $${CR}_{der}={ADD}_{der}\times {SF}_{der}$$

(10) $${CR}_{ing}={ADD}_{ing}\times {SF}_{ing}$$

(11) $$TCR={CR}_{inh}+{CR}_{der}+{CR}_{ing}$$

where the *RfD* was the reference dose (mg/kg/day), *SF* was the slope factor ((mg/kg/day)^−1^), values of *RfD* and *SF* were shown in Table 4, Table 5.

**Table 4:** The toxicity parameters of heavy metals.

heavy metal	*SF*_*ing*_	*SF*_*inh*_	*SF*_*der*_	*RfD*_*ing*_	*RfD*_*inh*_	*RfD*_*der*_
As	1.5	4.30 × 10^−3^	1.5	3.00 × 10^−4^	1.23 × 10^−4^	3.00 × 10^−4^
Cd	6.1	1.80 × 10^−3^	6.1	1.00 × 10^−3^	1.00 × 10^−3^	1.00 × 10^−5^
Cr	–	42	–	3.00 × 10^−3^	2.86 × 10^−5^	6.00 × 10^−5^
Cu	–	–	–	4.00 × 10^−2^	4.00 × 10^−2^	1.20 × 10^−2^
Hg	–	–	–	3.00 × 10^−4^	1.50 × 10^−5^	8.60 × 10^−5^
Ni	–	8.40 × 10^−1^	–	2.00 × 10^−2^	2.06 × 10^−2^	5.40 × 10^−3^
Pb	8.50 × 10^−3^	4.20 × 10^−2^	1.70 × 10^−1^	3.50 × 10^−3^	3.52 × 10^−3^	5.25 × 10^−4^
Zn	–	–	–	3.00 × 10^−1^	3.00 × 10^−1^	6.00 × 10^−2^

Note: The units for *SF* and *RfD* are (mg/kg/day)^−1^, (mg/kg/day), respectively.

**Table 5:** The toxicity parameters of PAHs.

PAHs	*SF*_*ing*_	*SF*_*inh*_	*SF*_*der*_	*RfD*_*ing*_	*RfD*_*inh*_	*RfD*_*der*_
BaA	7.30 × 10^−1^	3.10 × 10^−1^	1.46	–	–	–
BkF	7.30 × 10^−2^	3.10 × 10^−2^	1.46 × 10^−1^	–	–	–
BbF	7.30 × 10^−1^	1.46	3.85 × 10^−1^	–	–	–
Chr	7.30 × 10^−3^	3.10 × 10^−3^	1.46 × 10^−2^	–	–	–
DahA	7.30	3.10	1.46 × 10	–	–	–
IcdP	7.30 × 10^−1^	3.10 × 10^−1^	1.46	–	–	–
NaP	–	–	–	4.00 × 10^−2^	8.57 × 10^−4^	2.00 × 10^−2^
Acy	–	–	–	6.00 × 10^−2^	3.00 × 10^−2^	3.00 × 10^−2^
Ace	–	–	–	6.00 × 10^−2^	3.00 × 10^−2^	3.00 × 10^−2^
Flu	–	–	–	4.00 × 10^−2^	2.00 × 10^−2^	2.00 × 10^−2^
Phe	–	–	–	3.00 × 10^−2^	1.50 × 10^−2^	1.50 × 10^−2^
Ant	–	–	–	3.00 × 10^−2^	1.50 × 10^−1^	1.50 × 10^−1^
Flt	–	–	–	4.00 × 10^−2^	2.00 × 10^−2^	2.00 × 10^−2^
Pyr	–	–	–	3.00 × 10^−2^	1.50 × 10^−2^	1.50 × 10^−2^
BghiP	–	–	–	3.00 × 10^−2^	1.50 × 10^−2^	1.50 × 10^−2^

Note: The units for *SF* and *RfD* are (mg/kg/day)^−1^ and (mg/kg/day), respectively.

### 2.4 Pathogenic microbial health risk assessment

#### 2.4.1 Hazard identification

The quantitative microbiological risk assessment (QMRA) model is a robust framework utilized to evaluate the magnitude of health risks in populations exposed to pathogenic microorganisms across various environmental media [57]. The QMRA process is traditionally structured into four distinct stages as outlined by the National Research Council in 1983: pathogen identification, exposure assessment, dose-response analysis, and risk characterization. In the context of this study, the pathogenic microbial health risk of TPMDC anode compost was evaluated based on the QMRA model, and main pathogenic microorganisms selected by dose-response relationships in the microbial risk assessment guideline-pathogenic microorganisms with focus on food and water [58]. The microorganisms under consideration included *Escherichia_coli*, *Salmonell*a, *Vibrio_cholerae*, *Legionella,* and *Shigella*.

#### 2.4.2 Exposure assessment

In this study, both occupational exposure and public exposure to pathogenic microorganisms were taken into account. The occupational exposure pathways included carriers (transport vehicles, mechanical equipment, etc.), soil, and bioaerosols [59]. These pathways are significant as they represent direct and frequent points of contact for workers in the field. Conversely, public exposure pathways mainly included incidental soil and aerosol exposure, where the impact of pathogens in aerosols was negligible due to 100 m away [60], therefore, incidental exposure to aerosols at the emission source is calculated.

##### 2.4.2.1 Exposure of germ-bearing objects

It was assumed that 0.1 g sludge per day contaminated the machineries and equipment, such as trucks and solid fertilizer distributors, forming pathogen carriers. For a single exposure, without considering the inactivation of pathogenic microorganisms on the carriers over time, and direct contact was main occupational exposure to sludge compost. Exposure of germ-bearing objects was calculated as Eq (12).

(12) fc=rc×ft

where *fc* was the concentration of sludge compost on the carriers, CFU/day, *rc* was the concentration of pathogens in sludge compost, CFU/g, *ft* was the amount of sludge compost transferred to the carriers, 0.1 g/day.

##### 2.4.2.2 Exposure of contaminated soil

The level of pathogens in the soil after sludge compost amendment was considered only once for exposure, and there was no need to calculate the decay with time, which was calculated as Eq (13).

(13) sc=rc×dr

where *sc* was the concentration of pathogens in the soil, CFU/g, *dr* was the soil dilution ratio, setting as 1.75 × 10^−3^ (dry basis) [61].

##### 2.4.2.3 Exposure to contaminated aerosols

At the sludge compost application site, pathogen microbial aerosolization rates were estimated using a ratio of 1:1000, and the ratio was used to estimate the initial pathogen microbial aerosol concentration in the air at the source of contamination. An empirical model [60] was used for the downwind pathogenic microbial aerosol transport model assuming a wind speed of 2.2 m/s, as shown in equation (14):

(14) ac=[(−0.0022×dis)+0.1849]×16.6

where *ac* was aerosol pathogen concentration, CFU/m^3^, *dis* was windward distance from the source 2∼100m.

##### 2.4.2.4 Human exposure dose estimation

The equation for estimating the exposure dose for direct exposure to pollutants was shown in equation (15).

(15) d=ec×ft×hm×ds

where *d* was the pathogen dose, CFU/day, *ec* was the pathogen concentration in each individual exposure route, CFU/g, *ft* was the contaminant transfer rate to hand, *hm* was the contaminant transfer rate from hand to mouth, setting at 43 % and 36 %, respectively [62]. *ds* was the exposure to each individual exposure route (occupational exposure dose was 0.48 g/day and public incidental exposure was 0.05 g/day, which were estimated by USEPA, 1997) [63].

The exposure dose for aerosol exposure was estimated using the following Eq (16).

(16) d=(ec×br×t)×ag

where *br* was the respiration rate, 0.83 m^3^/h, *t* was the exposure time (8 h per day for occupational exposure and 1 h per day for the public), *ag* was 50 % aerosol uptake rate [64], *d* and *ec* had the same meaning as equation (15), and the results were shown in Fig. 7.

#### 2.4.3 Dose-response model

*Legionella* obeyed the first-order exponential dose-response model and the rest obeyed the *β*-Poisson model, the calculation equations according to Eq (17), (18), and the values of *α*, *β*, *γ* were shown in Table 6.

**Table 6:** The model parameters for quantitative health risk assessment of pathogenic microorganisms.

	pathogenic microorganism name				
	Escherichia_coli	Selenomonas	Legionella	Shigella	Vibrio_cholerae
*α*	0.1778	0.1324	–	0.21	0.25
*β*	1780000	51.45	–	42.86	16.2
*r*	0.1778	0.1324	–	0.21	0.25

##### 2.4.3.1 *β*-Poisson model

(17) $$P_{I}=1-{\left[1+\left(\frac{d}{\beta }\right)\right]}^{-\alpha }$$

where *P*_*I*_ was the probability of infection based on a single pathogen exposure, *d* was the pathogen dose as calculated in Eq (15), (16), *α* and *β* were the constant describing the dose-response curve.

##### 2.4.3.2 First-order exponential dose-response model

(18) $$P_{I}=1-\exp\left(-\gamma d\right)$$

where *P*_*I*_ was the probability of infection based on a single pathogen exposure, *γ* was the probability of a single pathway capable of causing disturbance, *d* was the pathogen dose (in equivalent doses) [58].

## 3 Results and discussions

### 3.1 Electricity production performance

**Fig. 2:**
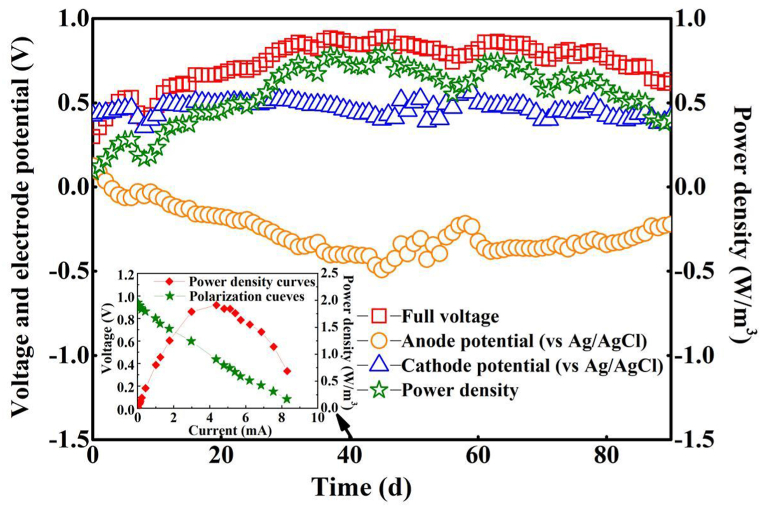
Power generation performance in TPMDC.

The power generation characteristics of the TPMDC system during the whole operation were depicted in Fig. 2. The system exhibited a prolonged and stable operation after a 4-days successful initiation period, sustaining a full voltage in excess of 0.7 V for a continuous duration of 65 days. This result aligns with the findings reported by Cao et al. [65]. On the 45th day, the system achieved its peak performance, with a full voltage of 0.893 V and a volumetric power density of 0.797 W/m^3^, reaching a maximum power density of 1.910 W/m^3^ when the internal resistance was measured at 104.7 Ω (Fig. 2a). These results were significantly higher than those of other solid-phase MFCs, such as plant-based, soil-based, and compost-based MFCs, with maximum voltages of 0.6, 0.52, and 0.75 V, respectively, and maximum power densities of 0.37, 0.68, and 2.12 W/m^3^, respectively [33,66,67]. Furthermore, the TPMDC system reached a full voltage of 0.508 V by the fourth day, which represents a shorter start-up time than the biocathode MFC with sewage sludge as single substrate [68], This suggests that the organic matter present in kitchen waste is more readily accessible to electrogenic microorganisms than that found in sludge. Consequently, the anode substrate conditioned with kitchen waste demonstrates superior performance in terms of electricity generation.

### 3.2 Composting performance

**Fig. 3:**
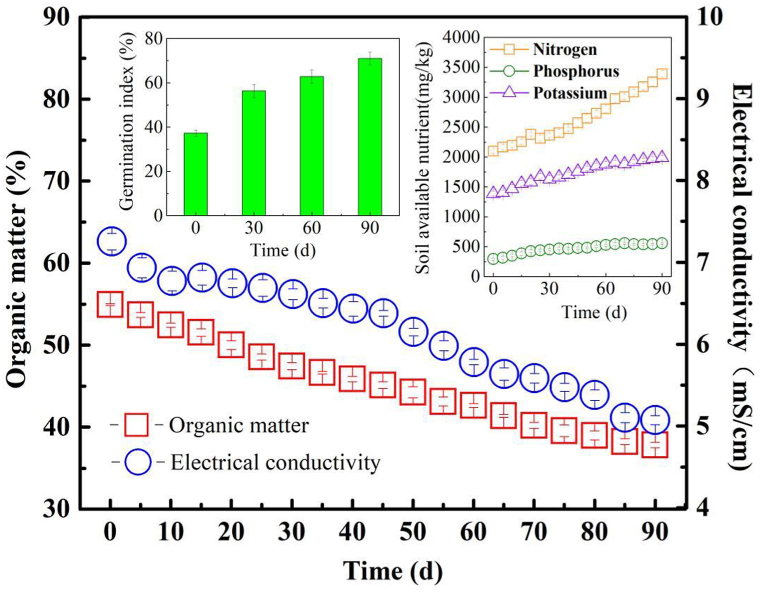
Composting performance in TPMDC.

**Fig. 4:**
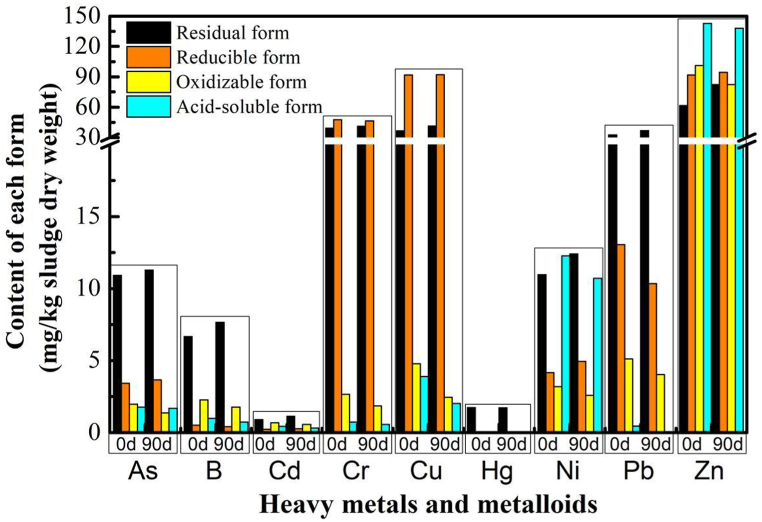
Changes in heavy metals or metalloids forms of anode substrates.

Throughout the operational cycle, the change of the organic matter, EC, and the availability of nutrients in the anode substrate were meticulously studied, as illustrated in Fig. 3. Concurrently, the heavy metal forms (Fig. 4), seed germination (Fig. 3), and pathogen abundance were integral to evaluating the composting performance of TPMDC system.

#### 3.2.1 Degradation of organic matters

The long-term power generation of the TPMDC system benefited from continuous degradation of abundant organic matters in the mixed anode substrate. The content of organic matters decreased from 54.93 % to 37.83 % over 90 days of operation, and a faster degradation occurred during the first 30 days of operation with a removal rate of 13.67 %, and the removal rate of organic matters reached 31.13 % at the end of operation, which was higher than that of 25.71 ± 0.15 % in the MDC with single dewatered sludge as anode substrate [43]. The final organic matter content in the anode compost could meet the requirement of higher than 10 % for soil amendment.

#### 3.2.2 Removal of salt

Based on the anode desalting function of TPMDC, EC in the anode substrate was reduced from 7.253 ± 0.102 mS/cm to 5.073 ± 0.057 mS/cm, and the salt removal rate reached 30.02 ± 0.04 % at the end of operation. The persistent reduction in EC levels within the anode substrate in TPMDC was fundamentally attributed to the operation of the concentration chamber, which facilitate the migration of soluble cations from the anode substrate towards the concentration chamber, driven by the bioelectric field, effectively accomplishing the goal of anode desalination. The EC of the anode substrate is a crucial determinant of the system's internal resistance and its capacity for power generation. Nonetheless, the introduction of kitchen waste, which is inherently rich in salts, resulted in a markedly higher salt content in the anode substrate compared to that of dewatered sludge. Despite this, the elevated salt levels did not substantially impair the system's power generation capabilities and they did present challenges for the subsequent utilization of the land. Throughout the operation, while the EC did not meet the standards for agricultural use (0.4–1 mS/cm), the process did reveal distinct development potential in terms of desalination compared to the original mixture of dewatered sludge and kitchen waste. Therefore, it is necessary to further improve the desalination performance of the anode substrate.

#### 3.2.3 Release of available nutrients

The available nutrients in sludge mainly referred to plant essential nutrients, such as available nitrogen (N), phosphorus (P), and potassium (K). During the degradation and mineralization processes of organic nitrogen and phosphorus within the mixed substrate, there is a concomitant increase in the availability of these nutrients. The content of available nitrogen increased from 2102.11 ± 37.02 mg/kg to 3389.45 ± 53.09 mg/kg, and that of available phosphorus increased from 295.22 ± 19.33 mg/kg to 557.13 ± 12.35 mg/kg. Notably, the concentration of available phosphorus was significantly lower than that of available nitrogen. The funding attributed to differential mineralization rates and uptake mechanisms. The content of available potassium also increased from 1388.31 ± 37.92 mg/kg to 1988.37 ± 39.02 mg/kg. The elevation of available potassium could be attributed to several key biological and chemical processes occurring within the mixed matrix. The role of potassium-solubilizing bacteria is paramount, as these microbes enhance the solubility of potassium through the secretion of organic acids and chelation, thereby increasing its availability to plants. Concurrently, potassium ions could migrate through the cation exchange membrane to the concentration chambers under the influence of the bioelectric field. This migration results in a relatively slow increase in potassium ion concentration during the later stages of operation. The mineralization of nitrogen, phosphorus, and potassium in the anode substrate was similar to the effects of anaerobic digestion and aerobic composting [69]. Consequently, TPMDC enhanced sludge degradation system could not only augments the availability of these essential nutrients in dewatered sludge but also promotes its suitability for land use [70].

#### 3.2.4 Reduced bioavailability of heavy metals

The alteration of heavy metal species in anode substrate serves as a pivotal indicator of compost quality. This study compared the forms transformation of heavy metals and metalloid in the anode substrate, both prior to and subsequent to a 90-day period of microbial electrochemically enhanced composting (as depicted in Fig. 4). In this composting system, nine heavy metals or metalloids (As, B, Cd, Cr, Cu, Hg, Ni, Pb, Zn) were identified, each present in four forms: acid-soluble, oxidizable, reducible, and residual states. The aggregate quantity of each heavy metal or metalloid across these four forms was found to be largely consistent before and after the composting operation. However, the residual state all increased except for Hg after 90 days of operation. This illustrated that the bioavailability of heavy metals or metalloid was significantly reduced after the 90-day composting period. These results were consistent with the study of sewage sludge high-temperature anaerobic digestion [71]. Especially, the bioavailability of Zn (reducible state) was elevated after composting, but it still was far below the relevant standards for organic solid waste land use. Thus, the study's outcomes underscore that the dewatered sludge and kitchen waste mixture, when subjected to composting enhancement by the TPMDC system, effectively mitigates the risk associated with heavy metal contamination.

#### 3.2.5 Increased seed germination

Seed germination index was an important index to judge the maturity and plant toxicity of organic fertilizer. After 30 days of microbial electrochemical compost, the seed germination index was close to 60 %, reaching the degree of basic maturity, and the seed germination index was more than 70 % after 90 days. These results would be beneficial for the further land use of dewatered sludge and kitchen waste mixed compost.

#### 3.2.6 Decreased pathogen abundance

Based on the microbial metagenomic analysis, the total abundance of pathogenic microorganisms in the original dewatered sludge was 1.248 %. After the addition of kitchen waste and TPMDC operation for 90 days, the total abundance of pathogenic microorganisms in the anode substrate was 1.108 %, suggesting a reduction in the overall risk of pathogenic microorganisms. Especially, the abundance of five pathogen species for risk prediction, *Escherichia_coli*, *Salmonella*, *Vibrio_cholerae*, *Legionella,* and *Shigella* were 0.012, 0.006, 0.001, 0.056, and 0.001 %, respectively.

### 3.3 Health risk assessment of heavy metals and PAHs

**Fig. 5:**
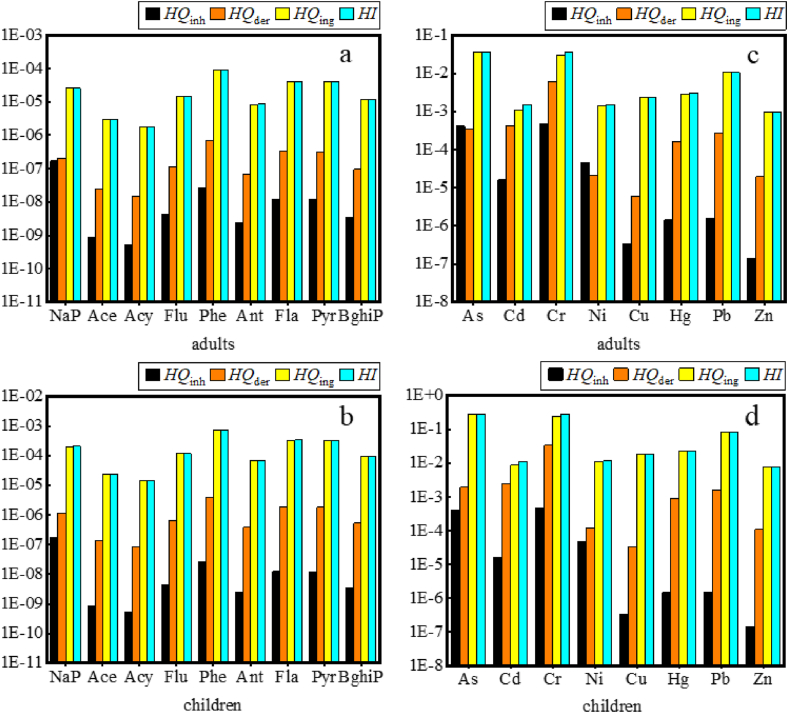
The *HQ* and *HI* values of heavy metals and PAHs for adults and children. (a) the *HQ* and *HI* values for PAHs for adults; (b) the *HQ* and *HI* values for PAHs for children; (c) the *HQ* and *HI* values for heavy metals for adults; (d) the *HQ* and *HI* values for heavy metals for children.

**Fig. 6:**
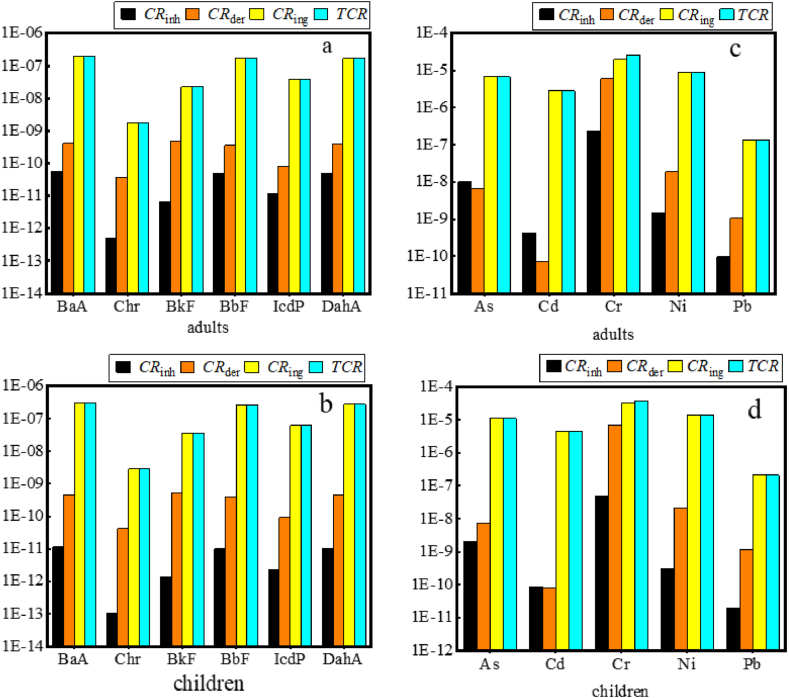
The *CR* and *TCR* values of heavy metals and PAHs for adults and children. (a) the *CR* and *TCR* values for PAHs for adults; (b) the *CR* and *TCR* values for PAHs for children; (c) the *CR* and *TCR* values for heavy metals for adults; (d) the *CR* and *TCR* values for heavy metals for children.

Heavy metals and PAHs were recognized as persistent environmental pollutants. Composting could transform the valence and forms of heavy metals [72,73], but did not reduce the total contents of heavy metals in the mixed compost substrate. Consequently, health risk assessment of mixed compost land use was of particularly important. This study conducted an evaluation of the health risk levels posed by soil heavy metals and PAHs following the land use of mixed compost for both adults and children. Table 2 showed the concentrations of heavy metals and PAHs in the amended soil, by which the *HQ*, *HI*, *CR*, and *TCR* values for heavy metals and PAHs were calculated by Eq (1) to Eq (11) for both adults and children, and the results were shown in Fig. 5, Fig. 6.

Through ingestion exposure, the total *HQ*_*ing*_ for heavy metals for adults and children reached up to 8.47 × 10^−2^ and 6.77 × 10^−1^, respectively, wherein As and Cr were the dominant contributors. For both adults and children, As contributed 38.46 % and 40.39 %, while Cr contributed 40.08 % and 37.58 %, respectively. The total *HQ*_*ing*_ for PAHs for adults and children was up to 2.36 × 10^−4^ and 1.89 × 10^−3^, respectively. Phenanthrene (Phe) was the major contributor to *HQ*_*ing*_ in both adults and children with 17.56 % and 37.93 %, respectively. In the case of dermal contact exposure, the total *HQ*_*der*_ of heavy metals were 7.33 × 10^−3^ and 4.11 × 10^−2^ for adults and children, respectively. Cr was the biggest contributor to the total *HQ*_*der*_, accounting for 82.81 % of the total *HQ*_*der*_ of heavy metals in adults, and the same phenomenon was observed in children. The total *HQ*_*der*_ of PAHs for adults and children were 1.88 × 10^−6^ and 1.06 × 10^−5^, respectively. The biggest contributor to the total *HQ*_*der*_ remained the Phe with the same contribution rate of 37.93 % in adults and children. For inhalation exposure, the *HQ*_*inh*_ for each heavy metal and PAH was respectively below 4.69 × 10^−4^ and 1.74 × 10^−7^ for both adults and children. From the above, the *HQ* values of children were always higher than that of adults in the three exposure routes, hence it was necessary to pay more attentions to the risk assessment of children during the land use of mixed compost. In addition, the total non-carcinogenic *HI* values for heavy metals and PAHs were all smaller than 1 in adults and children, with 9.29 × 10^−2^, 2.38 × 10^−4^ and 7.19 × 10^−1^, 1.90 × 10^−3^, respectively. This indicated that there was no significant risk of non-carcinogenic effects, wherein the proportion of *HQ*_*ing*_ was the highest, accounting for more than 90 %, indicating that ingestion was the overwhelmingly dominant exposure route, which is consistent with previous research reports [17,18].

The carcinogenic risks (*CR*) for four heavy metals (As, Cd, Cr, Ni, Pb) and six PAHs (BaA, Chr, BkF, BbF, IcdP, DahA) were individually evaluated for adults and children, with the results presented in Fig. 6. The total *TCR* values for the three exposure routes for adults and children were not exceeded the acceptable or tolerable risk level 10^−4^, with 4.51 × 10^−5^ and 6.89 × 10^−5^, respectively, and the biggest *TCR* values was Cr with 3.81 × 10^−5^ in children. These fundings indicated that soil amendment with dewatered sludge and kitchen waste mixed compost did not cause adverse health effects. Nonetheless, continuous vigilance is warranted due to the potential for cumulative impacts. Additionally, under the three exposure routes, the *CR* through ingestion (*CR*_*ing*_) was overwhelmingly dominant, with the proportions of 86.06 % and 90.10 % in adults and children, respectively, and merits heightened attention.

### 3.4 Health risk assessment of pathogenic microorganisms

Dewatered sludge from wastewater treatment plants is often contained a large number of pathogenic microorganisms [20], and the risk assessment of pathogenic microbial exposure during the land use of compost product was essential. This study employed a dose-response model to evaluate the infectious probability of pathogenic microorganisms in both occupational and public exposure scenarios, with the outcomes graphically represented in Fig. 8. In occupational exposure, the infectious probability was highest for *Legionella*, registering a value of 1.00, while the lowest was observed for *Escherichia_coli*, with a significantly lower value of 3.39 × 10^−6^ among the five pathogens examined. In the public population exposure, the infectious probability of *Legionella* was 2.16 × 10^−1^, and that of *Escherichia_coli* was 8.67 × 10^−8^. These findings underscore a substantially higher risk of infection for occupationally exposed individuals compared to the general public. Occupational exposure was characterized by prolonged contact times, multiplicity of exposure pathways, and potentially high concentrations of pathogens, culminating in a heightened risk of infection. However, this risk could be reduced through the implementation of effective occupational health measures, such as the use of gloves and protective masks. On the other hand, the characteristics of public exposure were brief exposure periods, infrequent contact, and lower pathogen concentrations, resulting in a minimal risk of infection per encounter. Viable measures include avoiding their presence in the work site during the mixed compost product application, which could reduce the direct exposure. Nonetheless, the risks identified in this study are specific to the exposure scenarios examined and do not account for a multitude of unforeseen circumstances. Limitations arise from an insufficient examination of pathogenic species, as well as unknown variables such as pathogen virulence, exposure frequency and duration, and population immunity levels. Consequently, the study acknowledges its inherent limitations and calls for more comprehensive research in the future to address these gaps.

**Fig. 7:**
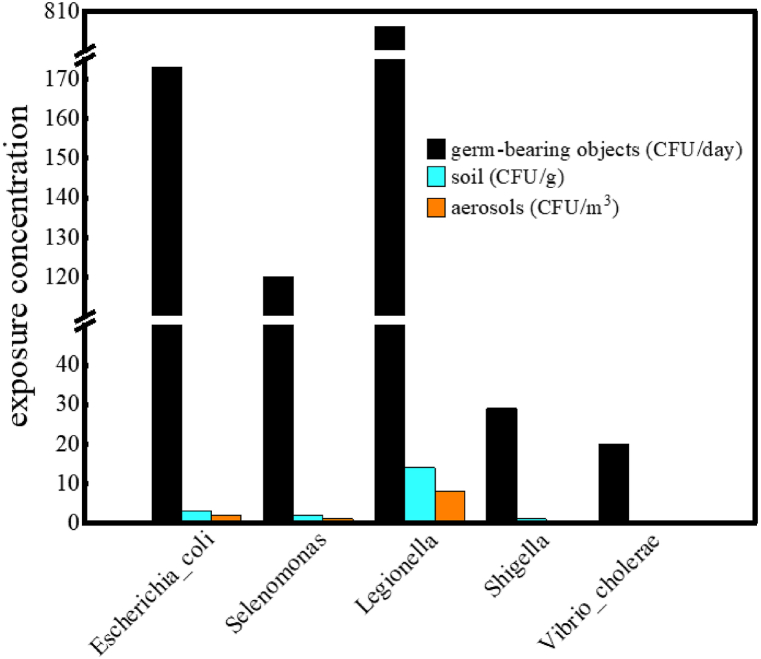
Exposure concentration of pathogenic microorganisms in various way.

**Fig. 8:**
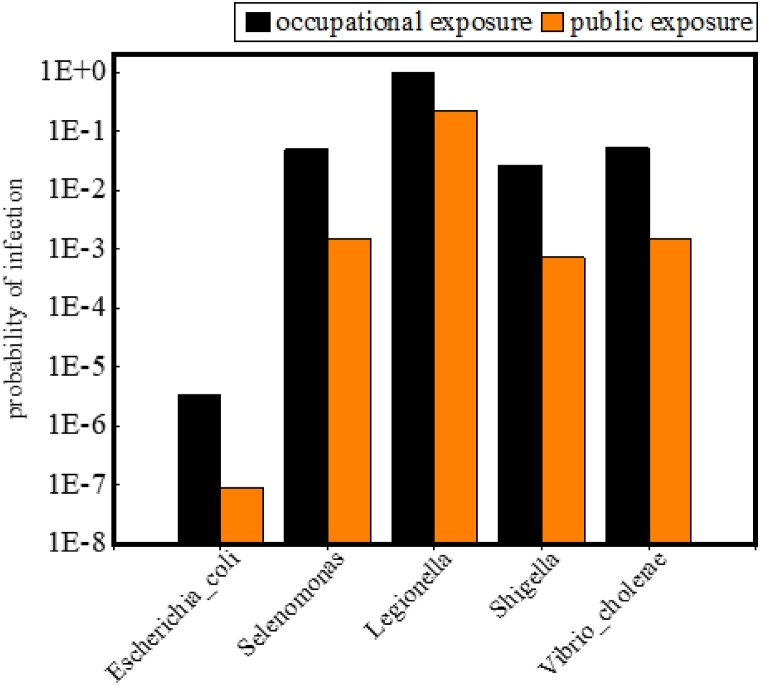
Probability of infection in occupational exposure and public exposure to one pathogenic exposure.

## 4 Conclusions

This study demonstrated the feasibility of co-composting of dewatered sludge and kitchen waste enhanced by the TPMDC, as well as an acceptable health risks associated with the land use of the compost products. Specifically, our results offered a viable means of recovering electrical energy with a maximum full voltage was 0.893 ± 0.005 V and a maximum volumetric power density was 0.797 ± 0.009 W/m^3^. The organic matters and salinity in mixes composted substrate were removed by 31.13 % and by 30.02 ± 0.04 %, respectively. The content of available nutrients increased significantly, while the bioavailability of heavy metals reduced. The seed germination index was improved significantly. The *HI* for heavy metals and PAHs in the amended soil was less than 1, indicating no significant non-cancer health risks. The *TCR* for heavy metals and PAHs were also within the acceptable risk level, with a *TCR* value less than 10^−4^. The risk values for the five pathogenic microorganisms examined were in the range of 8.67 × 10^−8^ to 1, confirming a low risk of infection. This study, therefore, holds significant scientific and practical importance, contributing valuable insights into the resourceful utilization of organic solid waste and advocating for the integration of microbial electrochemical systems in waste management strategies.

## Data availability statement

All the relevant data are included in the manuscript and the supplementary document. No separate repository is attached.

## CRediT authorship contribution statement

**Tengteng Hu:** Writing – original draft, Methodology, Data curation, Conceptualization. **Yunhan Lin:** Writing – review & editing. **Yingyu Liu:** Writing – review & editing. **Qingliang Zhao:** Writing – review & editing. **Hang Yu:** Writing – review & editing. **Zhugen Yang:** Writing – review & editing, Validation, Supervision. **Fanyu Meng:** Writing – review & editing, Writing – original draft, Validation, Supervision, Funding acquisition, Conceptualization.

## Declaration of competing interest

The authors declare that they have no known competing financial interests or personal relationships that could have appeared to influence the work reported in this paper.

## References

[1] Yan Y., Liu F., Gao J. (2022). Enhancing enzyme activity via low-intensity ultrasound for protein extraction from excess sludge. Chemosphere.

[2] Li M., Li F., Zhou J. (2022). Fallen leaves are superior to tree pruning as bulking agents in aerobic composting disposing kitchen waste. Bioresour. Technol..

[3] Huang H.J., Yuan X.Z. (2016). The migration and transformation behaviors of heavy metals during the hydrothermal treatment of sewage sludge. Bioresour. Technol..

[4] Haldar D., Shabbirahmed A.M., Singhania R.R. (2022). Understanding the management of household food waste and its engineering for sustainable valorization-a state-of-the-art review. Bioresour. Technol..

[5] Oleszczuk P. (2008). Phytotoxicity of municipal sewage sludge composts related to physico-chemical properties, PAHs and heavy metals. Ecotoxicol. Environ. Saf..

[6] Ramírez W.A., Domene X., Ortiz O. (2008). Toxic effects of digested, composted and thermally-dried sewage sludge on three plants. Bioresour. Technol..

[7] Barrera I., Andrés P., Alcañiz M.J. (2001). Sewage sludge application on soil: effects on two earthworm species. Water Air Soil Pollut..

[8] Domene X., Alcañiz M.J., Andrés P. (2006). Ecotoxicological assessment of organic wastes using the soil collembolan folsomia candida. Appl. Soil Ecol..

[9] Oleszczuk P. (2008). Heterocypris incongruens as a tool to estimate sewage sludge toxicity. Environ. Toxicol. Chem..

[10] Li M., Song G., Liu R. (2022). Inactivation and risk control of pathogenic microorganisms in municipal sludge treatment: a review. Front. Environ. Sci. Eng..

[11] Steele J.C., Meng X.Z., Venkatesan A.K. (2022). Comparative meta-analysis of organic contaminants in sewage sludge from the United States and China. Sci. Total Environ..

[12] Duan B., Zhang W., Zheng H. (2017). Comparison of health risk assessments of heavy metals and as in sewage sludge from wastewater treatment plants (WWTPs) for adults and children in the urban district of Taiyuan, China. Int. J. Environ. Res. Publ. Health.

[13] Tytła M., Widziewicz K., Zielewicz E. (2016). Heavy metals and its chemical speciation in sewage sludge at different stages of processing. Environ. Technol..

[14] Tytła M. (2019). Assessment of heavy metal pollution and potential ecological risk in sewage sludge from municipal wastewater treatment plant located in the most industrialized region in Poland-case study. Int. J. Environ. Res. Publ. Health.

[15] Bai Y., Shi K., Yu H. (2022). Source apportionment of polycyclic aromatic hydrocarbons (PAHs) in a sediment core from Lake Dagze Co, Tibetan Plateau, China: comparison of three receptor models. J. Environ. Sci. (China)..

[16] Smith S.R. (2009). Organic contaminants in sewage sludge (biosolids) and their significance for agricultural recycling. Philos Trans A Math Phys. Eng. Sci..

[17] Kim K.H., Jahan S.A., Kabir E. (2013). A review of airborne polycyclic aromatic hydrocarbons (PAHs) and their human health effects. Environ. Int..

[18] Rezaei Kalantary R., Jaafarzadeh N., Rezvani Ghalhari M. (2021). Cancer risk assessment of polycyclic aromatic hydrocarbons in the soil and sediments of Iran: a systematic review study. Rev. Environ. Health.

[19] Barbosa F Jr, Rocha B.A., Souza M.C.O. (2023). Polycyclic aromatic hydrocarbons (PAHs): updated aspects of their determination, kinetics in the human body, and toxicity. J. Toxicol. Environ. Health B Crit. Rev..

[20] Strauch D. (1991). Survival of pathogenic micro-organisms and parasites in excreta, manure and sewage sludge. Rev. Sci. Tech..

[21] Viau E., Bibby K., Paez-Rubio T. (2011). Toward a consensus view on the infectious risks associated with land application of sewage sludge. Environ. Sci. Technol..

[22] Wigginton R.K., Ye Y., Ellenberg M.R. (2015). Emerging investigators series: the source and fate of pandemic viruses in the urban water cycle. Environ. Sci. Water Res. Technol..

[23] Guo Q., Dai X. (2017). Analysis on carbon dioxide emission reduction during the anaerobic synergetic digestion technology of sludge and kitchen waste: taking kitchen waste synergetic digestion project in Zhenjiang as an example. Waste Manag..

[24] Shi Y., Deng Y., Wang G. (2020). Stackelberg equilibrium-based eco-economic approach for sustainable development of kitchen waste disposal with subsidy policy: a case study from China. Energy.

[25] Yang F., Li Y., Han Y. (2019). Performance of mature compost to control gaseous emissions in kitchen waste composting. Sci. Total Environ..

[26] Cukjati N., Zupančič G.D., Roš M. (2012). Composting of anaerobic sludge: an economically feasible element of a sustainable sewage sludge management. J. Environ. Manag..

[27] Liu S., Zhu N., Li L.Y. (2012). The one-stage autothermal thermophilic aerobic digestion for sewage sludge treatment: stabilization process and mechanism. Bioresour. Technol..

[28] Shen S., Chen Y., Zhan L. (2018). Methane hotspot localization and visualization at a large-scale Xi'an landfill in China: effective tool for landfill gas management. J. Environ. Manag..

[29] Tomei M.C., Rita S., Mininni G. (2011). Performance of sequential anaerobic/aerobic digestion applied to municipal sewage sludge. J. Environ. Manag..

[30] Chen Z., Li Y., Peng Y. (2022). Feasibility of sewage sludge and food waste aerobic co-composting: physicochemical properties, microbial community structures, and contradiction between microbial metabolic activity and safety risks. Sci. Total Environ..

[31] Wang H., Miao Z., Li Y. (2019). Energetically self-sustaining treatment of swine wastewater in a microbial electrochemical technology-centered hybrid system. Environ. Sci. Water Res. Technol..

[32] Yu H., Jiang J., Zhao Q. (2015). Bioelectrochemically-assisted anaerobic composting process enhancing compost maturity of dewatered sludge with synchronous electricity generation. Bioresour. Technol..

[33] Wang C.T., Liao F.Y., Liu K.S. (2013). Electrical analysis of compost solid phase microbial fuel cell. Int. J. Hydrogen Energy.

[34] Md Khudzari J., Tartakovsky B., Raghavan G.S.V. (2016). Effect of C/N ratio and salinity on power generation in compost microbial fuel cells. Waste Manag..

[35] Al-Mamun A., Ahmad W., Baawain S.M. (2018). A review of microbial desalination cell technology: configurations, optimization and applications. J. Clean. Prod..

[36] Luo H., Jenkins P.E., Ren Z. (2011). Concurrent desalination and hydrogen generation using microbial electrolysis and desalination cells. Environ. Sci. Technol..

[37] Jacobson K.S., Drew D.M., He Z. (2011). Efficient salt removal in a continuously operated up-flow microbial desalination cell with an air cathode. Bioresour. Technol..

[38] Mehanna M., Saito T., Yan J. (2010). Using microbial desalination cells to reduce water salinity prior to reverse osmosis. Energy Environ. Sci..

[39] Luo H., Xu P., Roane T.M. (2012). Microbial desalination cells for improved performance in wastewater treatment, electricity production, and desalination. Bioresour. Technol..

[40] Meng F., Zhao Q., Zheng Z. (2018). Simultaneous sludge degradation, desalination and bioelectricity generation in two-phase microbial desalination cells. Chem. Eng. J..

[41] Zhang R., El-Mashad H.M., Hartman K. (2007). Characterization of food waste as feedstock for anaerobic digestion. Bioresour. Technol..

[42] Awasthi M.K., Awasthi S.K., Wang Q. (2018). Role of Ca-bentonite to improve the humification, enzymatic activities, nutrient transformation and end product quality during sewage sludge composting. Bioresour. Technol..

[43] Meng F., Jiang J., Zhao Q. (2014). Bioelectrochemical desalination and electricity generation in microbial desalination cell with dewatered sludge as fuel. Bioresour. Technol..

[44] Sosnowski P., Klepacz-Smolka A., Kaczorek K. (2008). Kinetic investigations of methane co-fermentation of sewage sludge and organic fraction of municipal solid wastes. Bioresour. Technol..

[45] Cabbai V., Ballico M., Aneggi E. (2013). BMP tests of source selected OFMSW to evaluate anaerobic codigestion with sewage sludge. Waste Manag..

[46] Kumari M., Chandel M.K. (2023). Anaerobic Co-digestion of sewage sludge and organic fraction of municipal solid waste: focus on mix ratio optimization and synergistic effects. J. Environ. Manag..

[47] Logan B.E., Hamelers B., Rozendal R. (2006). Microbial fuel cells: methodology and technology. Environ. Sci. Technol..

[48] Bao S.D. (2000). Soil Science Society of China Beijing.

[49] Yang Y., Wang G.Y., Li G.X. (2021). Selection of sensitive seeds for evaluation of compost maturity with the seed germination index. Waste Manage. (Tucson, Ariz.).

[50] Chou J.D., Wey M.Y., Chang S.H. (2009). Evaluation of the distribution patterns of Pb, Cu and Cd from MSWI fly ash during thermal treatment by sequential extraction procedure. J. Hazard Mater..

[51] Ministry of ecology and environment of the People’s Republic of China. Soil and sediment–determination of polycyclic aromatic hydrocarbon by gas chromatography-mass spectrometry method (HJ 805 - 2016). https://www.mee.gov.cn/ywgz/fgbz/bz/bzwb/jcffbz/201606/W020160701543192146985.pdf.

[52] Luo X.S., Ding J., Xu B. (2012). Incorporating bioaccessibility into human health risk assessments of heavy metals in urban park soils. Sci. Total Environ..

[53] Ministry of ecology and environment of the People’s Republic of China. Technical guidelines for risk assessment of soil contamination of land for construction (HJ 25.3 - 2019). https://www.mee.gov.cn/ywgz/fgbz/bz/bzwb/trhj/201912/W020191224560850148092.pdf.

[54] Zhao X.G., Duan X.L. (2014). https://www.researchgate.net/publication/294645806_zhongguorenqunbaolucanshushouce_gaiyao.

[55] Zhao X.G., Duan X.L. (2016). https://www.researchgate.net/publication/308360758_zhongguorenqunbaolucanshushouceertongjuan_6-17sui_Exposure_Factors_Handbook_of_Chinese_Population_Children_6-17_years.

[56] Wang B.B., Duan X.L. (2016). https://www.researchgate.net/publication/308360736_zhongguorenqunbaolucanshushouceertongjuan_05sui_Exposure_Factors_Handbook_of_Chinese_Population_Children_0-5_years.

[57] WHO (2016). https://www.who.int/publications/i/item/9789241565370.

[58] Environmental Protection Agency (EPA) (2012). Microbial risk assessment guideline: pathogenic microorganisms with focus on food and water. https://www.epa.gov/sites/default/files/2013-09/documents/mra-guideline-final.pdf.

[59] Brooks J.P., McLaughlin M.R., Gerba C.P. (2012). Land application of manure and class B biosolids: an occupational and public quantitative microbial risk assessment. J. Environ. Qual..

[60] Brooks J.P., Tanner B.D., Josephson K.L. (2005). A national study on the residential impact of biological aerosols from the land application of biosolids. J. Appl. Microbiol..

[61] Gale P. (2005). Land application of treated sewage sludge: quantifying pathogen risks from consumption of crops. J. Appl. Microbiol..

[62] Rusin P., Maxwell S., Gerba C. (2002). Comparative surface-to-hand and fingertip-to-mouth transfer efficiency of gram-positive bacteria, gram-negative bacteria, and phage. J. Appl. Microbiol..

[63] USEPA (2012). https://www.epa.gov/expobox/about-exposure-factors-handbook.

[64] Medema G., Wullings B., Roeleveld P. (2004). Risk assessment of Legionella and enteric pathogens in sewage treatment works. Water Supply.

[65] Cao X., Huang X., Liang P. (2009). A new method for water desalination using microbial desalination cells. Environ. Sci. Technol..

[66] Huang D., Zhou S., Chen Q. (2011). Enhanced anaerobic degradation of organic pollutants in a soil microbial fuel cell. Chem. Eng. J..

[67] Khudzari M.J., Kurian J., Gariépy Y. (2018). Effects of salinity, growing media, and photoperiod on bioelectricity production in plant microbial fuel cells with weeping alkaligrass. Biomass Bioenergy.

[68] Zhang G., Zhao Q., Jiao Y. (2012). Efficient electricity generation from sewage sludge using biocathode microbial fuel cell. Water Res..

[69] Sánchez Ó.J., Ospina D.A., Montoya S. (2017). Compost supplementation with nutrients and microorganisms in composting process. Waste Manag..

[70] Hermassi M., Dosta J., Valderrama C. (2018). Simultaneous ammonium and phosphate recovery and stabilization from urban sewage sludge anaerobic digestates using reactive sorbents. Sci. Total Environ..

[71] Dąbrowska L., Rosińska A. (2012). Change of PCBs and forms of heavy metals in sewage sludge during thermophilic anaerobic digestion. Chemosphere.

[72] Huang Z.Y., Xie H., Cao Y.L. (2014). Assessing of distribution, mobility and bioavailability of exogenous Pb in agricultural soils using isotopic labeling method coupled with BCR approach. J. Hazard Mater..

[73] Zhu N.M., Li Qiang, Guo X.J. (2014). Sequential extraction of anaerobic digestate sludge for the determination of partitioning of heavy metals. Ecotoxicol. Environ. Saf..

